# Colon Lipoma Causing Colo-Colic Intussusception in an Adult: A Case Report from Tanzania

**DOI:** 10.1155/2024/7777258

**Published:** 2024-01-06

**Authors:** Jamil Suleiman, Mujaheed Suleman, Alex Mremi, Adnan Sadiq, Abbas Mohamedali, Dennis Machaku, Jay Lodhia

**Affiliations:** ^1^Department of General Surgery, Kilimanjaro Christian Medical Center, P.O. Box 3010, Moshi, Tanzania; ^2^Faculty of Medicine, Kilimanjaro Christian Medical University College, P.O. Box 2240, Moshi, Tanzania; ^3^Department of Pathology, Kilimanjaro Christian Medical Center, P.O. Box 3010, Moshi, Tanzania; ^4^Department of Radiology, Kilimanjaro Christian Medical Center, P.O. Box 3010, Moshi, Tanzania

## Abstract

Intussusception is rarely seen in adulthood but is commonly seen in the pediatric age group. Causes of intussusception in adults are commonly due to tumors and inflammatory diseases. Intussusception in adults accounts for less than 5% of intestinal obstruction. Colonic lipomas are usually asymptomatic and are mostly managed surgically with promising outcomes as seen in our case.

## 1. Introduction

Intussusception is commonly seen in children but rarely seen in adulthood and is often secondary to tumors, motility disorders, or inflammatory diseases [1]. Intussusception in adults accounts for approximately 1% of all bowel obstructions [1]. Colonic lipomas are usually asymptomatic and are found incidentally during autopsy, colonoscopy, or surgery, but larger ones may cause symptoms like bleeding, constipation, or pain [2]. Larger lipomas may be difficult to differentiate from malignancy before surgery; hence, diagnosis can be made from histological evaluation [2, 3]. Herein, we present an uncommon case of colonic lipoma causing colo-colic intussusception in an adult.

## 2. Case Presentation

A 57-year-old female presented to our outpatient surgical clinic with a one-month history of left lower abdominal pain, which was colicky in nature, nonradiating, and associated with nonprojectile bilious vomiting with episodes of bloodstained mucoid stools. There was no history of abdominal distension, abdominal surgery, or weight loss. She had visited several health centers with no relief of symptoms despite medical therapy. She denied a history of weight loss and cancers in her family.

Upon arrival, she was not pale, mildly dehydrated, not jaundiced with vitals within normal range and saturating at 98% on room air. Her abdominal examination revealed a nondistended abdomen with symmetrical abdominal contours, no mass nor organomegaly with 4 bowel sounds heard per minute on auscultation. A digital rectal examination revealed an anal tag at the 6 o'clock position, a normal anal tone, and no mass palpated. The gloved finger was stained with a copious amount of bloody stained mucoid stool.

**Figure 1:**
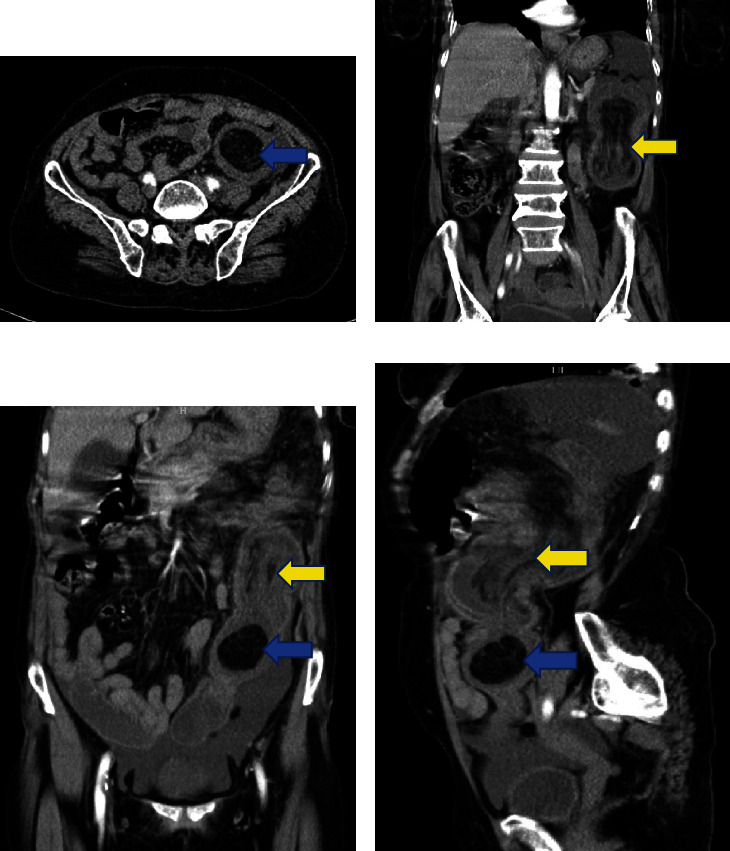
CT scan showing intussusception due to colon lipoma. Contrasted abdomen and pelvis CT. Axial (a), coronal (b, c), and sagittal (d) views show intussuscepted transverse colon (intussusceptum) with the mesenteric fat and vessels telescoped into the lumen of the descending colon (intussuscipiens) as shown in yellow arrows. The lead point is a lipoma (blue arrows), which is clearly demonstrated distally. Free fluid in the abdomen.

Her lab results showed a leucocyte count of 11.39 × 10^9^/L, hemoglobin of 11.2 g/dL, platelet of 583 × 10^9^/L, creatinine of 52 *μ*mol/L, and normal liver enzymes. Her abdominal CT scan revealed a target sign at the descending colon suggestive of colo-colic intussusception measuring 11.6 × 5.2 × 5.3 cm with a lipoma measuring 3.7 × 4.3 × 3.5 cm as a lead point (Figure 1). She was admitted and was taken for an emergency laparotomy, whereby a colo-colic intussusception with a 6 × 5 cm lipomatous intraluminal mass in the distal transverse colon was found with an edematous colon and its mesentery. The intussusception was successfully reduced manually, then the mass with a 5 cm negative margin of the transverse colon was resected, and a transverse double-barrel colostomy was raised successfully as the colon was not prepared to have fecal content within.

**Figure 2:**
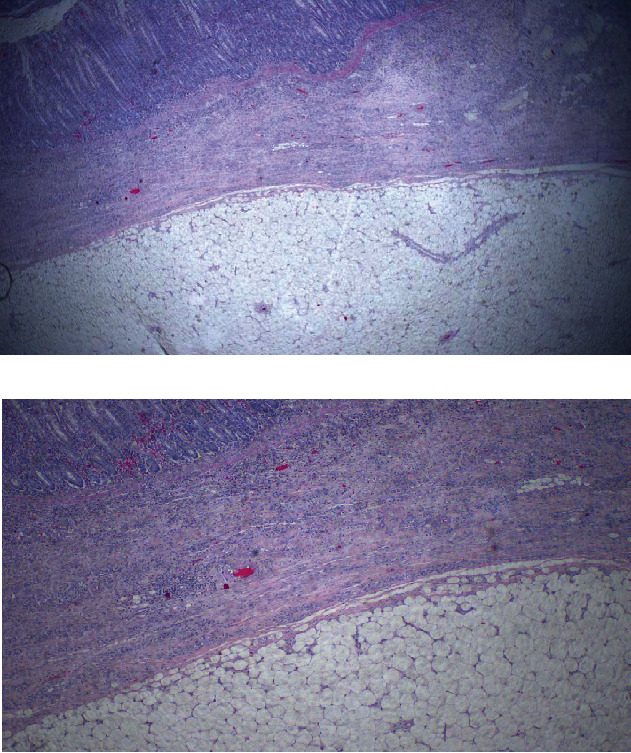
Photomicrographs showing colon lipoma histology. (a) Benign well-circumscribed colonic neoplasm composed of mature adipose tissue arising from submucosa with normal overlying mucosa. H&E stained low power (20x original magnification). (b) Histopathology of colonic lipoma. Intermediate magnification (40x original magnification).

Her postoperative recovery was uneventful and was discharged on day three postoperatively with instructions on colostomy care and to return to the surgical outpatient clinic for follow-up and wound care. Histology of the resected mass revealed a submucosal mass completely obstructing the large bowel lumen with microscopic features of reactive colon mucosa, a tumor composed of lobules of bland mature fat cells suggestive of a lipoma with negative margins (Figure 2).

**Figure 3:**
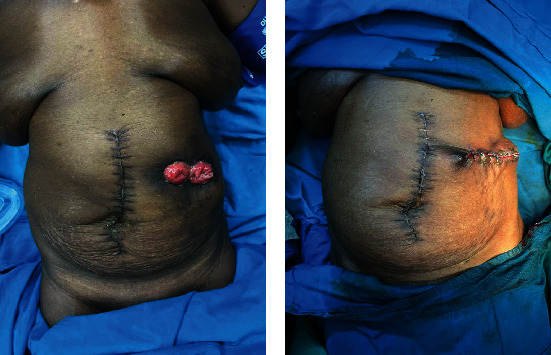
Pre- and postoperative clinical photographs. (a) Clinical photograph after first laparotomy showing double-barrel transverse colostomy. (b) Post colostomy closure (second surgery).

She was then reviewed in the outpatient clinic and scheduled for colostomy closure after 3 months. Her preoperative blood works were within normal range. Her previous incision had healed with a functioning transverse colostomy (Figure 3). Colostomy closure was done after thorough bowel preparation, was discharged two days later, and had an uneventful follow-up visit.

## 3. Discussion

Colon lipoma is a rare benign adipose tumor of the gastrointestinal tract, first described by Bauer in 1757 [4, 5]. The incidence ranges between 0.2 and 4.4% with a female predominance, frequently affecting women aged between 40 and 70 years [6–8]. Colonic lipomas typically arise from the submucosal area in approximately 90% of cases as in the index case, but may extend into the muscularis propria and 10% are subserosal, with sizes varying between 2 mm and 30 cm [5, 6, 9]. In an up-to-date systematic review in 2021 by Menegon Tasselli et al., colonic lipoma causing colo-colic intussusception was predominantly localized in the transverse colon, followed by the sigmoid colon, the cecum, the ascending colon, the descending colon, and least commonly found in the rectum [3]. Our case was comparatively consistent with the literature with regard to the gender, age, and location of the lipoma.

Generally, they are small and asymptomatic, although 6%-25% of patients with colonic lipoma have symptoms, with abdominal pain being the most common, followed by constipation, rectal bleeding, and diarrhea [10, 11]. Adult intussusception is rare, constituting less than 5% of all intussusception cases, and a majority of them have a malignant etiology. Uncommon causes of intussusception include adenomas, polyps, endometriosis, and lipomas [1, 12]. Lipomas larger than 4 cm are considered giant lipomas and 88% of them cause colo-colic intussusception [3].

Computed tomography having a sensitivity of 71–87% and a specificity of up to 100% remains the preferred radiological modality for the diagnosis of intussusception [13, 14]. It allows for the clear identification of the intussuscepting tissue as well as the lead point, presenting the typical appearance known as the “target sign” or “sign of donut” as seen in our case [12, 15]. Studies have shown the use of MRI being superior due to its effectiveness in highlighting adipose lesions because of the peculiar characteristics of the signal intensity of this tissue, especially T1-weighted and fat-suppressed images [4, 16]. Colonoscopy has been used for direct vision of the lesion, biopsies, and therapeutic resection of lipomas with pedicles and diameters of less than 2 cm. Pathognomonic signs for lipoma on a colonoscopy include the “naked fat sign” or “bare fat mark sign” which is seen as leakage of fat after biopsy, “tenting sign” portraying a tent-like appearance once the covering mucosa is lifted with forceps, and “pillow mark sign” or “cushion sign” representing a soft lesion with a cushion-like mucosal indentation when pressed with closed biopsy forceps [4, 12, 17, 18]. In our case, intussusception was present and diagnosis of lipoma was established via a CT scan before the surgery; hence, an MRI or colonoscopy was not required.

The majority of authors advocate surgery as the standard method of treatment for every colonic lipoma greater than 2 cm in size [1, 19], although surgical resection remains the treatment of choice and produces an excellent prognosis. Lipomas with a diameter smaller than 2 cm or pedunculated lipomas with a thin stalk can be resected endoscopically [14, 20]. Surgical treatment includes resection, colotomy with local excision, limited colon resection, segmental resection, hemicolectomy, or subtotal colectomy; the choice of the surgical interventions mainly depends on the size, location of the lipomas, and the presence or absence of definite preoperative diagnosis or disease complications [21]. Surgical excision is highly recommended in the event of intussusception and intestinal obstruction, especially in elderly patients, because of the high risk of underlying malignancy [22]. The prognosis depends on the complete removal of the tumor [23].

## 4. Conclusion

Colo-colic intussusception is a rare complication of a rarely occurring colon lipoma. CT imaging remains the method of choice for studying abdominal lipomas, particularly those rising into the layers of the colonic wall. Surgical resection remains the treatment of choice and produces excellent prognosis.
